# Electrical Impedance Tomography-Based Evaluation of Anesthesia-Induced Development of Atelectasis in Obese Patients

**DOI:** 10.3390/jcm13247736

**Published:** 2024-12-18

**Authors:** Stefanie Nothofer, Alexander Steckler, Mirko Lange, Anja Héžeľ, Christian Dumps, Hermann Wrigge, Philipp Simon, Felix Girrbach

**Affiliations:** 1 Anaesthesiology and Operative Intensive Care, Faculty of Medicine, University of Augsburg, 86156 Augsburg, Germany; stefanie.nothofer@uni-a.de (S.N.); alexander.steckler@uni-a.de (A.S.); christian.dumps@uk-augsburg.de (C.D.); philipp.simon3@uk-augsburg.de (P.S.); 2 Department of Anesthesiology and Intensive Care, University of Leipzig Medical Center, 04103 Leipzig, Germany; mirko.lange@medizin.uni-leipzig.de (M.L.); ahezel@online.de (A.H.); 3 Department of Anaesthesiology, Intensive Care and Emergency Medicine, Pain Therapy, Bergmannstrost Hospital Halle, 06112 Halle, Germany; hermann.wrigge@bergmannstrost.de; 4 Integrated Research and Treatment Centre Adiposity Diseases, University of Leipzig, 04103 Leipzig, Germany

**Keywords:** atelectasis, electrical impedance tomography, EIT, general anesthesia, intubation, lung ventilation, mechanical ventilation

## Abstract

**Background/Objectives:** The induction of general anesthesia leads to the development of atelectasis and redistribution of ventilation to non-dependent lung regions with subsequent impairment of gas exchange. However, it remains unclear how rapidly atelectasis occurs after the induction of anesthesia in obese patients. We therefore investigated the extent of atelectasis formation in obese patients in the first few minutes after the induction of general anesthesia and initiation of mechanical ventilation in the operating room. **Methods**: In 102 patients with morbid obesity (BMI ≥ 35 kg m^−2^) scheduled for laparoscopic intrabdominal surgery, induction of general anesthesia was performed while continuously monitoring regional pulmonary ventilation using electrical impedance tomography. Distribution of ventilation to non-dependent lung areas as a surrogate for atelectasis formation was determined by taking the mean value of five consecutive breaths for each minute starting five minutes before to five minutes after intubation. Ventilation inhomogeneity was assessed using the Global Inhomogeneity Index. **Results**: Median tidal volume in non-dependent lung areas was 58.3% before and 71.5% after intubation and increased by a median of 13.79% after intubation (*p* < 0.001). Median Global Inhomogeneity Index was 49.4 before and 71.4 after intubation and increased by a median of 21.99 units after intubation (*p* < 0.001). **Conclusions**: Atelectasis forms immediately after the induction of general anesthesia and increases the inhomogeneity of lung ventilation.

## 1. Introduction

Obesity is recognized as one of the most important health issues worldwide and currently affects up to 35% of the population in developed countries. According to the data of the World Obesity Federation, the prevalence of obesity nearly tripled among women and quadrupled in men between 1975 and 2022 [1]. According to current estimates, the number of obese people will further rise to around 4 million in 2035 [1]. Consequently, the number of surgical interventions requiring general anesthesia in patients with obesity is likely to continue rising.

The induction of general anesthesia leads to a cranial displacement of the abdominal organs and the diaphragm due to loss of muscle tone, resulting in compression of basal lung segments [2,3,4]. Particularly, in conjunction with the application of high oxygen concentrations, as used for preoxygenation during the induction of general anesthesia, this leads to the formation of resorption atelectasis [5].

In consequence, depending on various factors, up to 20% of the lung is collapsed in the basal sections after induction of general anesthesia in normal-weight patients. Although the development of atelectasis is observed to a variable extent in up to 90% of patients [3], the increased weight of the abdominal and thoracic adipose tissue in patients with obesity further increases the cranial displacement of the diaphragm and therefore the extent of atelectasis formation [6,7,8]. As the functional residual capacity (FRC) is already reduced in patients with obesity when they are awake [9], a further reduction in FRC and the ventilation–perfusion mismatch caused by atelectasis formation can intraoperatively result in a critical impairment of arterial oxygenation.

Furthermore, tidal recruitment, which refers to the cyclic opening and collapse of lung tissue, produces lung injury and is a possible cause for pulmonary and extrapulmonary postoperative complications [10]. It can thus be postulated that the greater extent of atelectasis formation in obese patients may significantly contribute to the markedly elevated incidence of postoperative complications observed in these patients [11].

Numerous studies have previously investigated different ventilation settings and maneuvers to reduce atelectasis formation and its consequences, but it is mostly unclear how rapidly atelectasis occurs after intubation and initiation of mechanical ventilation. A few previous computed tomography-based studies have demonstrated that atelectasis develops within the first minutes after intubation in obese patients [4,12]. Real-time data assessing the effect of mechanical ventilation, however, is lacking. One method to monitor regional lung ventilation and assess inhomogeneity of ventilation within the lung is electrical impedance tomography (EIT), a non-invasive approach allowing real-time bedside monitoring of pulmonary ventilation [13]. Several studies have used EIT to conduct studies assessing regional lung ventilation in different patient collectives as it presents an easily accessible, radiation-free technique compared to other radiographic imaging techniques [14,15,16,17,18,19].

In this secondary analysis of three prospective, randomized controlled trials, we hypothesized that the formation of atelectasis occurs within the first few minutes after the initiation of mechanical ventilation in obese surgical patients. To do so, EIT monitoring was performed continuously 5 min before and up to 5 min after induction of general anesthesia in obese patients scheduled for laparoscopic bariatric surgery and the ventral tidal volume (vV_t_) of the non-dependent lung as well as the GI index were calculated and compared before and after intubation.

## 2. Materials and Methods

We performed a secondary analysis of EIT data from three randomized controlled trials conducted at the University Hospital of Leipzig, Germany [11,17,18] (ClinicalTrials.gov, No. NCT02148692; German Clinical Trials Register, No. DRKS00004199, No. DRKS00013984). Written informed consent was obtained from all patients before inclusion in each respective study. Approval for all three studies was granted by the local institutional ethics committee of the University of Leipzig (No. 334/16-lk; No. 196-11-ff-8042011; No. 475/17-ek). A detailed description of the inclusion criteria and methods of the analyzed studies can be found in the published articles.

### 2.1. Patients

Inclusion criteria of all studies were adult obese patients with a BMI ≥ 35 kg m^−2^ scheduled for laparoscopic bariatric surgery and a medium to high risk for postoperative pulmonary complications (Assess Respiratory Risk in Surgical Patients in Catalonia [ARISCAT] score ≥ 26) [20]. For the present secondary analysis, we computed data from all patients of the original studies.

### 2.2. Electrical Impedance Tomography (EIT)

EIT is a non-invasive and radiation-free technique to assess regional lung ventilation in real time. To perform EIT, an elastic belt with 16 evenly spaced electrodes is placed around the thorax, and a small alternating current (1–10 mA) is repeatedly injected at a high frequency (50–80 kHz) through each pair of electrodes. Raw EIT data represent changes in impedance that result from a change in conductivity depending on the type of tissue in between the electrodes. After processing and rendering these data into a 32 × 32 two-dimensional matrix, an image representing the spatial distribution of impedance through the lung is created allowing for the assessment of ventilation in different lung regions [21]. Regions of interest (ROI) represent subdivisions of the lung and enable comparison of the ventilation in different parts of the lung. To assess the distribution of tidal ventilation to the dependent and non-dependent lung areas, the EIT image can be divided into four equally sized transversal ROIs. The ventral tidal volume corresponds to the proportion of the tidal volume in the two ventral ROIs (in per cent), compared to the total tidal volume in all four ROIs.

The Global Inhomogeneity (GI) Index is a quantitative, dimensionless measure quantifying the gas distribution of the tidal volume in the lung. Each pixel in the lung image generated by EIT represents impedance differences between end-expiration and end-inspiration. The GI Index is calculated by relating the sum of absolute deviations from these mean impedance changes across all pixels in the lung image to the total sum of impedance changes observed across the lung within the entire respiratory cycle [22]. A high GI Index indicates greater inhomogeneity of ventilation within the lung potentially due to conditions such as atelectasis formation. It is calculated as demonstrated below.

(1) $$GI=\frac{\sum_{x,y\in lung}\;\left|{DI}_{xy-}Median({DI}_{lung})\right|}{\sum_{x,y\in lung}{DI}_{xy}}$$

DI: differential impedance in tidal images; DI_xy_: pixel in the identified lung area; DI_lung_: all pixels represented in the lung area; ∈: element of the ventilated part of the lung [21].

### 2.3. EIT Measurements

EIT was performed with a commercially available EIT system (PulmoVista 500; Draeger Medical AG, Luebeck, Germany) in all studies. EIT files were recorded from the start of preoxygenation. The recording was stopped 5 min after intubation. Tidal distributions of ventilation to dependent and non-dependent lung regions and the Global Inhomogeneity Index were determined offline using the Draeger EIT analysis tool (version 6.1; Draeger Medical AG) as well as customized institutional software.

### 2.4. Induction of General Anesthesia and Mechanical Ventilation

Preoxygenation was performed in the ramped position at an F_I_O_2_ of 1.0 with zero end-expiratory pressure using either a tight-fitting face mask (n = 38) or a mouthpiece with nose clamp (n = 64) for 3–5 min. Induction of general anesthesia was performed using a continuous remifentanil infusion (0.1–0.5 µg/kg ideal body weight [IBW]; n = 38) or a bolus dose of sufentanil (0.2–0.3 µg/kg total body weight, n = 64) and standard doses of propofol (2–3 mg/kg adjusted body weight) and rocuronium (0.6 mg/kg IBW; n = 102). Maintenance of anesthesia was either performed as total intravenous anesthesia (remifentanil/propofol) or balanced anesthesia (sufentanil/remifentanil and sevoflurane or desflurane).

Mechanical ventilation settings in the first 5 min after intubation were set according to the protocols of the primary studies. In all patients, constant-flow, volume-controlled mechanical ventilation was provided by an intensive care ventilator (EVITA-XL; Draeger Medical AG, Germany) with a tidal volume (V_T_) of 7 mL/kg predicted body weight (n = 38), or 8 mL/kg predicted body weight (n = 64). F_I_O_2_ was set to 0.4 or higher if necessary to achieve a target oxygen saturation measured by pulse oximetry greater than or equal to 92% in all trials. The initial respiratory rate was set to 12 breaths per minute, and the inspiratory-to-expiratory ratio and inspiratory time were set to achieve end-inspiratory and end-expiratory zero flow with inspiratory pause. In line with the study protocols, PEEP was initially set to 4 cmH_2_O (n = 38) or 5 cmH_2_O (n = 64) after intubation.

### 2.5. Endpoints and Study Procedure

**Figure 1:**
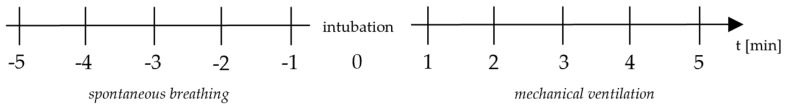
Demonstration of the study process using the EIT method. The Global Inhomogeneity Index and the ventral tidal volume were determined for five consecutive minutes pre- and post-intubation by taking the mean value of five consecutive breaths at each minute.

Primary endpoints are the ventral tidal volume (vV_T_) and, therefore, the percentage of the total tidal volume distributed to the non-dependent lung, and the GI Index during spontaneous breathing before intubation compared to after intubation and onset of mechanical ventilation. Both GI Index and vV_t_ were determined at each minute starting 5 min before intubation to 5 min after intubation and onset of mechanical ventilation, as demonstrated in Figure 1.

### 2.6. Statistical Analysis

Data are presented as median (95% confidence intervals) if not stated otherwise. Normal distribution was tested by the Shapiro–Wilk test and by plotting QQ plots but was not met for either endpoint. The vV_T_ and the GI Index were compared at each minute pre- and post-intubation with the Friedman test for non-normally distributed variables. Median vV_t_ and GI Index before and after intubation were calculated over all five measurements pre- and post-intubation and compared with the Wilcoxon signed-rank test. The vV_T_ and GI Index one minute after intubation were compared with the measurements 2, 3, 4, and 5 min after intubation by the Wilcoxon signed-rank test. Implausible (negative vV_t_, etc.) or missing data were excluded from analysis. All statistical analyses were performed using SPSS Version 29.0.0.0 (IBM SPSS Statistics for Windows, IBM, Armonk, NY, USA). All tests were two-tailed, and a *p* < 0.05 was considered statistically significant.

## 3. Results

**Figure 2:**
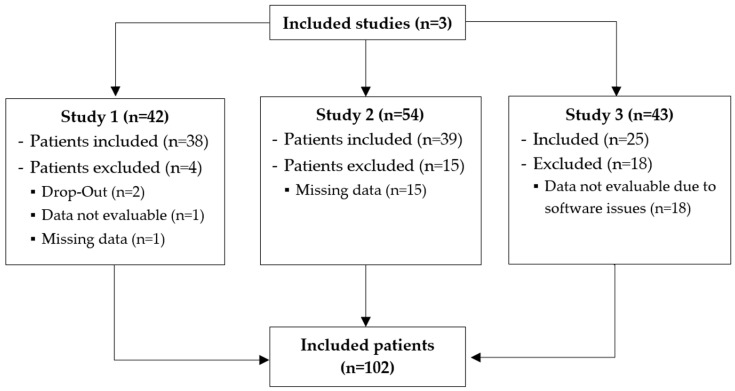
Flow chart of the included studies.

Data from a total of 102 patients were included in this analysis. A flow chart of the included patient data from the three randomized, controlled trials is demonstrated in Figure 2. In total, 65.7% of patients were female, and the mean body mass index across all three study populations was 49.9 ± 8.9 kg m^−2^. Baseline characteristics did not differ between the study populations of the three studies (Table 1).

### Distribution of Tidal Ventilation and GI Index Five Minutes Pre- and Post-Intubation

**Figure 3:**
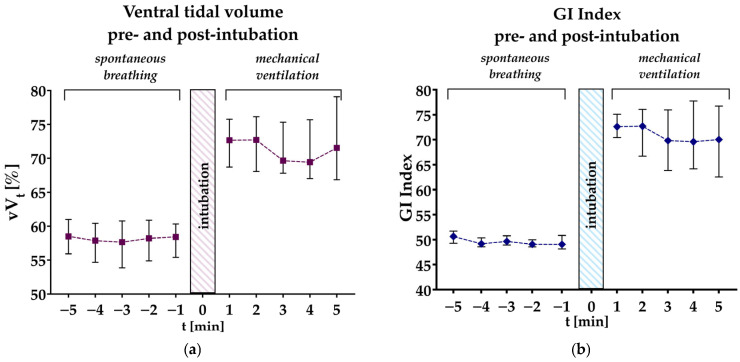
(**a**) Percentage of the ventral tidal volume (vV_T_) at each minute from five minutes before to five minutes after intubation. (**b**) Global Inhomogeneity Index (GI Index) from five minutes before to five minutes after intubation. Squares with whiskers represent median with 95% confidence intervals.

**Figure 4:**
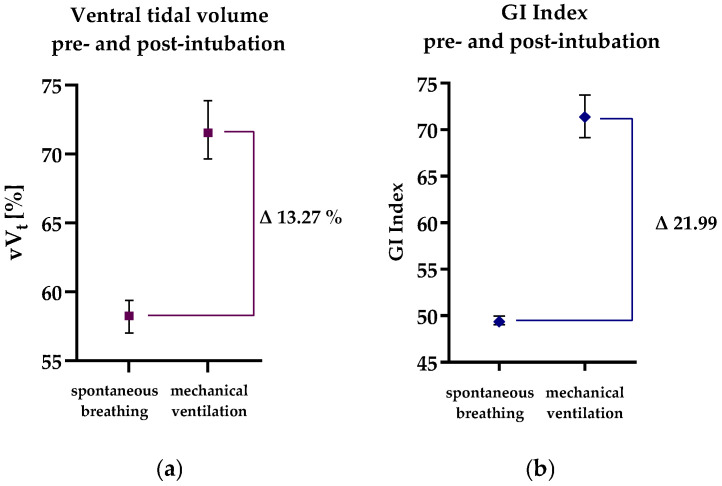
(**a**) Ventral tidal volume (vV_T_) and (**b**) Global Inhomogeneity Index (GI Index) before and after intubation. Squares with whiskers represent median and 95% confidence intervals.

Median distribution of the ventral tidal volume (vV_t_), expressed as the percentage of tidal volume in the non-dependent lung, at each minute from 5 min before to 5 min after intubation, ranged from 57.7% to 58.8% in spontaneously breathing patients but increased to 69.4% to 73.7% after intubation (*p* < 0.001) (see Table 2). The median vV_T_ calculated over the five measurements before and after intubation was 58.3% before and increased by 13.27% to 71.5% after intubation (*p* < 0.001) (see Table 3 and Figure 3 and Figure 4). The GI index calculated at each minute before intubation ranged from 49.1 to 50.6 before intubation, indicating only minimal ventilation inhomogeneity during spontaneous breathing. After intubation, the GI Index at each minute increased to 69.6 to 72.7, signifying greater inhomogeneity of regional ventilation within the lung (*p* < 0.001) (see Table 2). The median GI Index calculated over all five measurements was 49.4 before intubation and increased significantly by 22.0 to 71.4 after intubation (*p* < 0.001) (see Table 3 and Figure 3 and Figure 4). A pairwise comparison of the median vV_T_ one minute after intubation with the vV_T_ at minutes 2 (*p* = 0.638), 3 (*p* = 0.736), 4 (*p* = 0.775), and 5 (*p* = 0.852) showed that the vV_T_ did not change significantly after the first minute following intubation. The same applies to the GI Index one minute after intubation compared to the measurements at 2 (*p* = 0.950), 3 (*p* = 0.527), 4 (*p* = 0.819), and 5 (*p* = 0.370) minutes after intubation.

## 4. Discussion

This secondary analysis of EIT data from three randomized, prospective studies reveals four important aspects of atelectasis formation associated with the induction of general anesthesia in patients with morbid obesity. First, patients with morbid obesity already have a significant amount of atelectasis in dependent lung areas before the start of preoxygenation, even if the ramped position is used for anesthesia induction. Second, the induction of general anesthesia leads to a notable shift of V_T_ to non-dependent lung areas, indicating an additional, extensive atelectasis formation in this patient cohort. Third, we observed that atelectasis formation occurs rapidly after the induction of anesthesia and is already fully developed before the start of mechanical ventilation. Fourth, atelectasis formation is associated with a significant deterioration of ventilation homogeneity, which was indicated by a simultaneous rise in the GI index after intubation.

In a previous study of our working group including only normal-weight patients with a mean BMI of 25.3 kg m^−2^, mean tidal volume in non-dependent lung areas was ~52%, although the induction of anesthesia was performed in the supine position compared to the ramped position in the current study [16]. Nevertheless, the mean % V_T_ in non-dependent lung areas in the patients of the current study was 58%, suggesting greater atelectasis in obese patients who are already awake, even in the ramped position. These results are in line with a previous CT-based study from Eichenberger et al., who also found that, already before the induction of anesthesia, the amount of atelectasis in patients with morbid obesity was significantly higher and was about twice as high as in normal-weight patients [23]. Recently, Mancilla-Galindo et al. also described a high prevalence of atelectasis in awake obese patients. In their CT-based study, atelectasis percentage increased with higher BMI classes and was up to 10.46% in patients with a BMI > 50 kg m^−2^ [24].

Jones and colleagues found in their physiological study in awake patients that a rise in BMI of 5 kg m^−2^ is associated with a 5–15% reduction in functional residual capacity (FRC) [9]. In a previous study, which is also part of this secondary analysis, our working group was able to show using the nitrogen washout method that the already reduced FRC in obese patients in an awake state is reduced by a further 50% after the induction of general anesthesia. It has been known for many years that the development of atelectasis can lead to a significant reduction in oxygenation by increasing the ventilation–perfusion mismatch and the pulmonary shunt fraction [25,26]. However, this significant anesthesia-induced reduction in FRC and atelectasis formation could largely be reversed by a standardized recruitment maneuver with subsequent application of an individualized, EIT-guided PEEP [18].

Obese patients are exposed to a higher risk of developing atelectasis after initiation of mechanical ventilation due to several pathophysiological changes impairing normal lung physiology [26]. Essentially, two different mechanisms are discussed that contribute to the formation of atelectasis in obese patients. Excessive abdominal and thoracic adipose tissue increase compression forces to the lung and lead to a more pronounced cephalad displacement of the diaphragm [8] resulting in lower lung compliance [27] and a reduced functional residual capacity even before, but especially after general anesthesia induction in obese patients [7]. Furthermore, rapid closure of proximal airways in patients with obesity undergoing general anesthesia with consecutive resorption atelectasis is discussed. In this case, the time needed for the development of resorption atelectasis distal to the collapsed airways may be dependent on the fraction of inspired oxygen, with the shortest development time occurring with the high oxygen concentrations used during preoxygenation. There is a strong correlation between the susceptibility towards small airway collapse and an increase in the body mass index [28,29].

Based on our data, it is not possible to determine the underlying mechanism leading to the observed loss of aerated lung tissue (i.e., central vs. more peripheral airway collapse). However, the clinical significance of this theoretical distinction still has to be determined. If the loss of aerated lung tissue was attributed to an initial collapse of more central airways with secondary development of resorption atelectasis, the application of higher PEEP values that are sufficient enough to re-open collapsed central airways immediately after intubation should prevent the development of larger resorption atelectasis distal to the collapsed airways.

Post-induction atelectasis formation and airway closure was also associated with an increase in ventilation inhomogeneity in our study, indicated by a significant rise in the GI index. In addition to the development of atelectasis, another effect could have contributed to an increase in GI Index. The direct compression of small airways in patients with morbid obesity results in the inhomogeneous distribution of airway resistance in the lung [30]. As recently discussed by De Jong et al. [31], inhomogeneous inflation and deflation of the lungs secondary to a significant heterogeneity in resistance and compliance in obese patients may lead to a pendelluft effect. These pendelluft effects are detectable by electrical impedance tomography and are also associated with an increase in GI Index [32].

As discussed above, early atelectasis formation in dependent lung areas after induction of anesthesia leads to a significant reduction in aerated lung volume. This in turn means that the set tidal volume is distributed over less lung tissue. Analogous to the “baby-lung” effect, the tidal volumes set in the current patient cohort could therefore have already led to relevant overinflation in non-dependent lung areas of some patients—even though, with a tidal volume of 8 mL/kg ideal body weight, they formally met the requirements for lung-protective ventilation. Early studies on the Global Inhomogeneity (GI) Index were already able to prove that regional hyperinflation is accompanied by an increase in the GI [33].

Our study has several limitations. First, we did not directly assess the extent of atelectasis by computed tomography or by the nitrogen washout method, so we were not able to determine the exact percentage of atelectatic lung tissue. However, in one of our previous studies, we observed that the distribution of tidal ventilation measured by EIT correlates sufficiently well with changes in end-expiratory lung volume [16]. Second, PEEP levels during our observation period were relatively low. Therefore, it remains unclear what the extent and dynamics of atelectasis formation would have been if higher PEEP values were set immediately after intubation.

As discussed above, lung volume is rapidly and extensively reduced in patients with morbid obesity immediately after the induction of anesthesia. This has important clinical implications. Based on our results, a ventilation strategy consisting of early recruitment of atelectatic lung tissue in combination with individualized positive end-expiratory pressure may be the preferred strategy to prevent hypoxia and secondary lung injury due to hyperinflation of non-atelectatic lung areas. Further clinical research should also focus on strategies to maintain positive airway pressure during anesthesia induction to limit airway collapse and to reduce the extent of early atelectasis formation in patients with morbid obesity. For example, it is possible that the use of non-invasive ventilation during the induction of anesthesia could be an effective strategy, as it was used in the PREOXI trial [34].

## 5. Conclusions

In this secondary analysis including patients with morbid obesity, we found an immediate shift of tidal ventilation to non-dependent lung areas after the induction of general anesthesia, suggesting that atelectasis formation rapidly occurs in these patients, even before the initiation of invasive mechanical ventilation. The shift of tidal ventilation to non-dependent lung areas was accompanied by a significant increase in ventilation inhomogeneity.

## Figures and Tables

**Table 1:** Baseline characteristics of the patient populations. Entries are given as mean ± standard deviation or numbers (percentage).

	Total	Study 1	Study 2	Study 3
Number of cases	102	38	39	25
Sex, female n (%)	67 (65.7)	26 (68.4)	25 (64.1)	16 (64.0)
Age [years]	45 ± 12.6	45 ± 13.6	47 ± 11.4	44 ± 12.7
Height [cm]	171.0 ± 12.1	169.4 ± 13.2	172.2 ± 10.9	170.5 ± 10.5
Weight [kg]	147.4 ± 33.5	147.6 ± 45.2	152.8 ± 21.8	142.8 ± 31.4
BMI [kg m^−2^]	49.9 ± 8.9	50.0 ± 11.9	51.8 ± 8.0	48.3 ± 9.1

**Table 2:** Comparison of the Global Inhomogeneity Index (GI Index) and the ventral tidal volume (vV_T_) at each minute from 5 min before (−5 to −1 min) to 5 min after intubation and initiation of mechanical ventilation (+1 to +5 min). Data are presented as median with 95% confidence intervals. N corresponds to the number of patients of whom the specific value was available. *p*-values were determined by the Friedman test. * significant *p*-value < 0.05.

	Before Intubation *Spontaneous Breathing*					After Intubation *Mechanical Ventilation*					Before vs. After Intubation
t (min)	−5	−4	−3	−2	−1	+1	+2	+3	+4	+5	*p*-Value *
% V_T_ non-dependent											
n	68	70	78	84	93	94	83	62	56	50	<0.001
median	58.5	57.8	57.7	58.2	58.4	72.7	72.7	69.7	69.4	71.5	
95% CI	55.9–61.0	54.7–60.5	53.8–60.8	54.9–60.9	55.4–60.3	68.7–75.8	68.1–76.1	67.8–75.3	67.0–75.7	66.9–79.1	
GI Index											
n	67	69	81	91	99	94	78	54	43	36	<0.001
median	50.6	49.2	49.7	49.1	49.1	72.6	72.7	69.8	69.6	70.0	
95% CI	49.3–51.7	48.6–50.4	48.9–50.8	48.5–50.0	48.1–50.9	70.4–75.1	66.7–76.1	63.8–76.0	64.2–77.8	62.6– 76.7	

**Table 3:** Comparison of the Global Inhomogeneity Index (GI Index) and the ventral tidal volume (vV_T_) before and after intubation. GI Index and vV_t_ are each calculated over all values pre- and post-intubation (median and 95% confidence intervals). Statistical analysis was performed with the Wilcoxon signed-rank test. * significant *p*-value < 0.05.

	Before Intubation Spontaneous Breathing	After Intubation Mechanical Ventilation	Δ	*p*-Value *
median vV_T_ (%)	58.26	71.53	+13.27%	<0.001
95% CI for vV_T_ (%)	57.02–59.39	69.66–73.88		
median GI Index	49.37	71.36	+21.99	<0.001
95% CI for GI Index	49.05–49.97	69.16–73.73		

## Data Availability

The data presented in this study are available from the corresponding author (F.G.), upon reasonable request.
